# 4-Bromo-3-methyl­anilinium perchlorate 18-crown-6 clathrate

**DOI:** 10.1107/S1600536811003540

**Published:** 2011-02-05

**Authors:** Qian Xu, Min Min Zhao

**Affiliations:** a Ordered Matter Science Research Center, College of Chemistry and Chemical, Engineering, Southeast University, Nanjing 211189, People’s Republic of China

## Abstract

The reaction of 4-bromo-3-methyl­anilinium perchlorate and 18-crown-6 in methanol solution yielded the title compound, C_7_H_9_BrN^+^·ClO_4_^−^·C_12_H_24_O_6_. The protonated 4-bromo-3-methyl­amine unit contains one –NH_3_^+^ substituent, resulting in a 1:1 supra­molecular rotator–stator structure, (C_7_H_9_Br—NH_3_^+^)(18-crown-6), through three bifurcated N—H⋯(O,O) hydrogen bonds between the ammonium group of the cation and the O atoms of the crown ether mol­ecule.

## Related literature

For the structures of similar crown ether clathrates, see: Akutagawa *et al.* (2002 ▶); Ge & Zhao (2010*a* ▶,*b* ▶;); Guo & Zhao (2010 ▶); Zhao (2010 ▶); Zhao & Qu (2010*a* ▶,*b* ▶). The title compound was prepared as part of a study of ferroelectric materials. For their properties, see: Fu *et al.* (2007 ▶); Zhang *et al.* (2009 ▶); Ye *et al.* (2009 ▶).
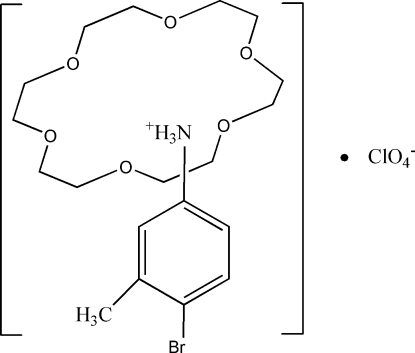


## Experimental

### 

#### Crystal data

C_7_H_9_BrN^+^·ClO_4_^−^·C_12_H_24_O_6_
*M* *_r_* = 550.81
Monoclinic, 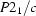

*a* = 11.967 (2) Å
*b* = 13.446 (3) Å
*c* = 15.677 (3) Å
β = 94.05 (3)°
*V* = 2516.3 (9) Å^3^
*Z* = 4
Mo *K*α radiation
μ = 1.79 mm^−1^
*T* = 296 K
0.40 × 0.30 × 0.20 mm

#### Data collection

Rigaku SCXmini diffractometer
Absorption correction: multi-scan (*CrystalClear*; Rigaku, 2005 ▶) *T*_min_ = 0.530, *T*_max_ = 0.699
25007 measured reflections
5665 independent reflections
4005 reflections with *I* > 2σ(*I*)
*R*_int_ = 0.066

#### Refinement

*R*[*F* ^2^ > 2σ(*F* ^2^)] = 0.060
*wR*(*F* ^2^) = 0.157
*S* = 1.09
5665 reflections
289 parameters
H-atom parameters constrained
Δρ_max_ = 0.41 e Å^−3^
Δρ_min_ = −0.98 e Å^−3^

### 

Data collection: *CrystalClear* (Rigaku, 2005 ▶); cell refinement: *CrystalClear*; data reduction: *CrystalClear*; program(s) used to solve structure: *SHELXS97* (Sheldrick, 2008 ▶); program(s) used to refine structure: *SHELXL97* (Sheldrick, 2008 ▶); molecular graphics: *SHELXTL* (Sheldrick, 2008 ▶); software used to prepare material for publication: *SHELXTL*.

## Supplementary Material

Crystal structure: contains datablocks I, global. DOI: 10.1107/S1600536811003540/jh2260sup1.cif

Structure factors: contains datablocks I. DOI: 10.1107/S1600536811003540/jh2260Isup2.hkl

Additional supplementary materials:  crystallographic information; 3D view; checkCIF report

## Figures and Tables

**Table 1:** Hydrogen-bond geometry (Å, °)

*D*—H⋯*A*	*D*—H	H⋯*A*	*D*⋯*A*	*D*—H⋯*A*
N1—H1*A*⋯O8	0.89	2.22	2.905 (4)	134
N1—H1*A*⋯O9	0.89	2.19	2.966 (4)	145
N1—H1*B*⋯O5	0.89	2.19	2.955 (4)	144
N1—H1*B*⋯O10	0.89	2.22	2.912 (4)	134
N1—H1*E*⋯O6	0.89	2.29	2.970 (4)	133
N1—H1*E*⋯O7	0.89	2.12	2.893 (4)	145

**Fig. 1.**
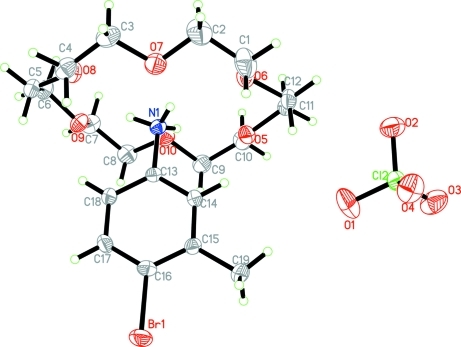
The title molecules with the atomic numbering scheme. The displacement ellipsoids are drawn at the 30% probability level.

## supplementary crystallographic information

### Comment

There is currently a great deal of interest in crown ethers because of their ability to form non-covalent, H-bonding complexes with ammonium cations both in solid and in solution (Akutagawa *et al.*, 2002; Ge *et al.*, 2010*a*,*b*; Guo *et al.*, 2010; Zhao *et al.*, 2010*a*,*b*). Not only the size of the crown ether, but also the nature of the ammonium cation (–NH_4_^+^, RNH_3_^+^, *R*_2_NH_2_^+^, *etc*) can influence on the stoichiometry and stability of these host–guest complexes. The host molecules combine with the guest species by intermolecular interaction, and if the host molecule possess some specific sites, it is easy to realise high selectivity in ion or molecular recognitions. 18-Crown-6 have the highest affinity for ammonium cation RNH_3_^+^, while most studies of 18-crown-6 and its derivatives invariably showed a 1:1 stoichiometry with RNH_3_^+^ cations.

Dielectric permittivity of the title compound is tested to systematically investigate the ferroelectric phase transitions materials (Ye *et al.*, 2009; Zhang *et al.*, 2009). The title compound has no dielectric anomaly with the value of 5 and 8 under 1*M* Hz in the temperature from 80 to 433 K (the compound m.p.> 453 K), suggesting that the compound should be no distinct phase transition occurred within the measured temperature range.

The title compound is composed of cationic [C_7_H_9_NBr(18-Crown-6)]^+^ and one single anionic [ClO_4_]^-^ anions (Fig. 1). Supramolecular rotators was assembled between protonated 4-bromo-3-methylanilinium [C_7_H_6_Br—NH_3_]^+^ and 18-crown-6 by hydrogen-bonding. The ammonium moieties of (–NH_3_^+^) cations were interacted with the oxygen atom of crown ethers through six simple N—H···O hydrogen bonding, forming 1:1 supramolecular rotator-stator structures.

The macrocycle adopts a conformation with approximate D_3 d_ symmetry, with all O—C—C—O torsion angles being *gauche* and alternating in sign, and all C—O—C—C torsion angles being *trans*. The C—N bonds of [C_7_H_6_Br—NH_3_]^+^ were almost perpendicular to the mean oxygen planes of crown ethers.

Supramolecular cation structure, [C_7_H_9_NBr(18-Crown-6)]^+^, were introduced as counter cations to [ClO_4_]^-^ anions. Cl has a flattened tetrahedral coordination by four O^2-^ ions [range of *cis*-bond angles = 108.4 (2)–110.3 (2) °; dav(Cl—O) = 1.426 (3)–1.457 (3) Å].

The title compound was stabilized by intramolecular N—H···O hydrogen bonds, but no intermolecular hydrogen bond was observed. The intramolecular N—H···O hydrogen bonding length are within the usual range: 2.893 (4) and 2.970 (4) Å.

### Experimental

C_7_H_8_NBr. HClO_4_ (2 mmol, 0.57 g) and 18-crown-6 (2 mmol, 0.528 g) were dissolved in 40 ml me thanol solution. The precipitate was filtered out. Two days later, single crystals suitable for X-ray diffraction analysis were obtained from slow evaporation of methanol solution at 0°C.

### Refinement

All the C—H hydrogen atoms were calculated geometrically, with C—H = 0.93, 0.97 and 0.96 Å for aromatic, methylene and methyl H respectively, and constrained to ride on their parent atoms with *U*_iso_(H) = *xU*_eq_(C), where *x* = 1.5 for methyl H and *x* = 1.2 for all other H atoms.

All the N—H hydrogen atoms were calculated geometrically. The positions of the H atoms of the nitrogen atoms were refined using a riding model with N—H = 0.89 Å and *U*_iso_(H) = 1.5*U*_eq_(N).

### Crystal data

**Table:**

C_7_H_9_BrN^+^·ClO_4_^−^·C_12_H_24_O_6_	*F*(000) = 1144
*M**_r_* = 550.81	*D*_x_ = 1.454 Mg m^−^^3^
Monoclinic, *P*2_1_/*c*	Mo *K*α radiation, λ = 0.71073 Å
Hall symbol: -P 2ybc	Cell parameters from 20001 reflections
*a* = 11.967 (2) Å	θ = 3.0–27.3°
*b* = 13.446 (3) Å	µ = 1.79 mm^−^^1^
*c* = 15.677 (3) Å	*T* = 296 K
β = 94.05 (3)°	Prism, colorless
*V* = 2516.3 (9) Å^3^	0.40 × 0.30 × 0.20 mm
*Z* = 4	

### Data collection

**Table:**

Rigaku SCXmini diffractometer	5665 independent reflections
Radiation source: fine-focus sealed tube	4005 reflections with *I* > 2σ(*I*)
graphite	*R*_int_ = 0.066
Detector resolution: 28.5714 pixels mm^-1^	θ_max_ = 27.3°, θ_min_ = 3.0°
CCD_Profile_fitting scans	*h* = −15→15
Absorption correction: multi-scan (*CrystalClear*; Rigaku, 2005)	*k* = −17→17
*T*_min_ = 0.530, *T*_max_ = 0.699	*l* = −20→20
25007 measured reflections	

### Refinement

**Table:**

Refinement on *F*^2^	Primary atom site location: structure-invariant direct methods
Least-squares matrix: full	Secondary atom site location: difference Fourier map
*R*[*F*^2^ > 2σ(*F*^2^)] = 0.060	Hydrogen site location: inferred from neighbouring sites
*wR*(*F*^2^) = 0.157	H-atom parameters constrained
*S* = 1.09	*w* = 1/[σ^2^(*F*_o_^2^) + (0.0769*P*)^2^ + 0.8528*P*] where *P* = (*F*_o_^2^ + 2*F*_c_^2^)/3
5665 reflections	(Δ/σ)_max_ = 0.001
289 parameters	Δρ_max_ = 0.41 e Å^−^^3^
0 restraints	Δρ_min_ = −0.98 e Å^−^^3^

### Special details

**Table:**

Geometry. All e.s.d.'s (except the e.s.d. in the dihedral angle between two l.s. planes) are estimated using the full covariance matrix. The cell e.s.d.'s are taken into account individually in the estimation of e.s.d.'s in distances, angles and torsion angles; correlations between e.s.d.'s in cell parameters are only used when they are defined by crystal symmetry. An approximate (isotropic) treatment of cell e.s.d.'s is used for estimating e.s.d.'s involving l.s. planes.
Refinement. Refinement of *F*^2^ against ALL reflections. The weighted *R*-factor *wR* and goodness of fit *S* are based on *F*^2^, conventional *R*-factors *R* are based on *F*, with *F* set to zero for negative *F*^2^. The threshold expression of *F*^2^ > σ(*F*^2^) is used only for calculating *R*-factors(gt) *etc*. and is not relevant to the choice of reflections for refinement. *R*-factors based on *F*^2^ are statistically about twice as large as those based on *F*, and *R*- factors based on ALL data will be even larger.

### Fractional atomic coordinates and isotropic or equivalent isotropic displacement parameters (Å^2^)

**Table:**

	*x*	*y*	*z*	*U*_iso_*/*U*_eq_	
Br1	0.19928 (4)	1.01458 (3)	−0.00712 (3)	0.06334 (18)	
O8	0.0452 (2)	0.60635 (19)	0.30049 (15)	0.0495 (6)	
N1	0.2459 (2)	0.72782 (19)	0.29922 (16)	0.0367 (6)	
H1A	0.1796	0.7195	0.3206	0.055*	
H1B	0.2937	0.7545	0.3389	0.055*	
H1E	0.2719	0.6692	0.2833	0.055*	
O9	0.0542 (2)	0.78578 (18)	0.39854 (15)	0.0499 (6)	
C15	0.3203 (3)	0.8828 (2)	0.1107 (2)	0.0370 (7)	
C16	0.2147 (3)	0.9217 (2)	0.08602 (19)	0.0398 (7)	
O6	0.4462 (2)	0.59829 (19)	0.29366 (17)	0.0551 (7)	
O5	0.4715 (2)	0.78411 (19)	0.37212 (16)	0.0518 (6)	
O7	0.2319 (2)	0.53548 (19)	0.21645 (17)	0.0554 (7)	
O10	0.2751 (2)	0.84908 (18)	0.45312 (16)	0.0506 (6)	
C13	0.2336 (3)	0.7946 (2)	0.22444 (19)	0.0332 (7)	
C17	0.1196 (3)	0.8976 (3)	0.1282 (2)	0.0453 (8)	
H17A	0.0503	0.9242	0.1098	0.054*	
C18	0.1292 (3)	0.8336 (2)	0.1982 (2)	0.0415 (8)	
H18A	0.0664	0.8171	0.2270	0.050*	
C14	0.3275 (3)	0.8187 (2)	0.1809 (2)	0.0398 (7)	
H14A	0.3966	0.7914	0.1990	0.048*	
C11	0.5624 (3)	0.7135 (3)	0.3751 (3)	0.0545 (10)	
H11A	0.5601	0.6721	0.4256	0.065*	
H11B	0.6335	0.7484	0.3781	0.065*	
C6	−0.0296 (3)	0.7105 (3)	0.4065 (2)	0.0537 (9)	
H6A	−0.0976	0.7405	0.4250	0.064*	
H6B	−0.0033	0.6619	0.4490	0.064*	
C10	0.4740 (3)	0.8459 (3)	0.4471 (3)	0.0600 (10)	
H10A	0.5406	0.8872	0.4499	0.072*	
H10B	0.4766	0.8045	0.4979	0.072*	
C7	0.0804 (3)	0.8360 (3)	0.4778 (2)	0.0562 (10)	
H7A	0.1013	0.7881	0.5224	0.067*	
H7B	0.0155	0.8726	0.4944	0.067*	
C19	0.4241 (3)	0.9087 (3)	0.0644 (3)	0.0580 (10)	
H19A	0.4878	0.8747	0.0911	0.087*	
H19B	0.4366	0.9792	0.0673	0.087*	
H19C	0.4132	0.8887	0.0056	0.087*	
C8	0.1762 (3)	0.9065 (3)	0.4666 (3)	0.0579 (10)	
H8A	0.1585	0.9496	0.4180	0.070*	
H8B	0.1891	0.9476	0.5172	0.070*	
C3	0.1271 (4)	0.4814 (3)	0.2176 (3)	0.0600 (10)	
H3A	0.1286	0.4386	0.2675	0.072*	
H3B	0.1164	0.4401	0.1670	0.072*	
C5	−0.0538 (3)	0.6604 (3)	0.3215 (2)	0.0517 (9)	
H5A	−0.1164	0.6149	0.3244	0.062*	
H5B	−0.0733	0.7096	0.2778	0.062*	
C4	0.0330 (3)	0.5545 (3)	0.2200 (2)	0.0539 (9)	
H4A	0.0356	0.6014	0.1731	0.065*	
H4B	−0.0383	0.5201	0.2144	0.065*	
C9	0.3713 (3)	0.9105 (3)	0.4438 (3)	0.0583 (10)	
H9A	0.3780	0.9592	0.4895	0.070*	
H9B	0.3633	0.9457	0.3897	0.070*	
C12	0.5517 (3)	0.6502 (3)	0.2967 (3)	0.0573 (10)	
H12A	0.5554	0.6914	0.2462	0.069*	
H12B	0.6128	0.6027	0.2978	0.069*	
C1	0.4329 (4)	0.5300 (4)	0.2234 (4)	0.0794 (15)	
H1C	0.4969	0.4856	0.2243	0.095*	
H1D	0.4290	0.5664	0.1698	0.095*	
C2	0.3278 (4)	0.4705 (3)	0.2295 (4)	0.0794 (15)	
H2A	0.3239	0.4185	0.1866	0.095*	
H2B	0.3280	0.4395	0.2854	0.095*	
Cl2	0.75937 (7)	0.70425 (7)	0.09959 (6)	0.0485 (2)	
O2	0.7570 (3)	0.6235 (2)	0.15986 (19)	0.0709 (8)	
O1	0.6613 (3)	0.7637 (3)	0.1044 (3)	0.0979 (12)	
O4	0.7607 (3)	0.6624 (3)	0.01394 (19)	0.0784 (9)	
O3	0.8582 (3)	0.7636 (3)	0.1177 (2)	0.0849 (10)	

### Atomic displacement parameters (Å^2^)

**Table:**

	*U*^11^	*U*^22^	*U*^33^	*U*^12^	*U*^13^	*U*^23^
Br1	0.0687 (3)	0.0700 (3)	0.0526 (3)	0.0157 (2)	0.0132 (2)	0.02676 (19)
O8	0.0416 (13)	0.0607 (16)	0.0458 (13)	−0.0055 (11)	−0.0003 (11)	−0.0053 (11)
N1	0.0388 (14)	0.0358 (14)	0.0359 (14)	−0.0015 (11)	0.0058 (11)	0.0020 (11)
O9	0.0502 (14)	0.0543 (15)	0.0469 (14)	−0.0092 (11)	0.0152 (11)	−0.0020 (11)
C15	0.0384 (17)	0.0343 (16)	0.0393 (17)	0.0015 (13)	0.0110 (14)	−0.0005 (13)
C16	0.052 (2)	0.0352 (17)	0.0332 (16)	0.0051 (14)	0.0053 (14)	0.0025 (13)
O6	0.0451 (14)	0.0589 (16)	0.0627 (16)	0.0068 (12)	0.0125 (12)	−0.0091 (13)
O5	0.0417 (14)	0.0535 (15)	0.0588 (16)	0.0012 (11)	−0.0066 (12)	0.0054 (12)
O7	0.0568 (16)	0.0443 (14)	0.0657 (17)	0.0002 (12)	0.0081 (13)	−0.0090 (12)
O10	0.0548 (15)	0.0390 (13)	0.0585 (16)	−0.0048 (11)	0.0074 (12)	−0.0054 (11)
C13	0.0372 (16)	0.0302 (15)	0.0326 (15)	−0.0003 (12)	0.0049 (13)	0.0004 (12)
C17	0.0337 (17)	0.053 (2)	0.050 (2)	0.0103 (15)	0.0032 (15)	0.0062 (16)
C18	0.0326 (17)	0.0468 (19)	0.0459 (18)	0.0020 (14)	0.0089 (14)	0.0067 (15)
C14	0.0338 (17)	0.0399 (18)	0.0461 (18)	0.0050 (14)	0.0054 (14)	0.0034 (14)
C11	0.0356 (19)	0.056 (2)	0.072 (3)	−0.0006 (16)	0.0029 (18)	0.019 (2)
C6	0.044 (2)	0.064 (2)	0.056 (2)	−0.0041 (17)	0.0191 (17)	0.0041 (18)
C10	0.053 (2)	0.050 (2)	0.073 (3)	−0.0102 (18)	−0.016 (2)	−0.007 (2)
C7	0.065 (3)	0.059 (2)	0.047 (2)	−0.0005 (19)	0.0175 (18)	−0.0075 (17)
C19	0.047 (2)	0.061 (2)	0.068 (3)	0.0070 (18)	0.0247 (19)	0.015 (2)
C8	0.066 (3)	0.051 (2)	0.058 (2)	0.0028 (19)	0.0092 (19)	−0.0115 (18)
C3	0.072 (3)	0.046 (2)	0.062 (3)	−0.0129 (19)	0.003 (2)	−0.0103 (18)
C5	0.0389 (19)	0.057 (2)	0.059 (2)	−0.0073 (17)	0.0030 (17)	0.0075 (18)
C4	0.059 (2)	0.058 (2)	0.045 (2)	−0.0166 (19)	−0.0031 (17)	−0.0041 (17)
C9	0.056 (2)	0.045 (2)	0.073 (3)	−0.0110 (18)	−0.004 (2)	−0.0066 (18)
C12	0.0360 (19)	0.073 (3)	0.064 (2)	0.0088 (18)	0.0142 (17)	0.013 (2)
C1	0.065 (3)	0.084 (3)	0.091 (4)	0.021 (2)	0.015 (3)	−0.035 (3)
C2	0.080 (3)	0.051 (3)	0.107 (4)	0.008 (2)	0.003 (3)	−0.032 (3)
Cl2	0.0381 (4)	0.0543 (5)	0.0526 (5)	0.0011 (4)	−0.0005 (4)	0.0077 (4)
O2	0.075 (2)	0.0661 (19)	0.0713 (19)	−0.0057 (15)	0.0047 (15)	0.0206 (15)
O1	0.074 (2)	0.105 (3)	0.114 (3)	0.043 (2)	0.002 (2)	−0.002 (2)
O4	0.080 (2)	0.101 (3)	0.0551 (18)	−0.0078 (19)	0.0068 (15)	−0.0074 (16)
O3	0.074 (2)	0.097 (2)	0.081 (2)	−0.0366 (19)	−0.0162 (17)	0.0177 (18)

### Geometric parameters (Å, °)

**Table:**

Br1—C16	1.921 (3)	C6—H6B	0.9700
O8—C4	1.440 (4)	C10—C9	1.503 (5)
O8—C5	1.448 (4)	C10—H10A	0.9700
N1—C13	1.476 (4)	C10—H10B	0.9700
N1—H1A	0.8900	C7—C8	1.507 (5)
N1—H1B	0.8900	C7—H7A	0.9700
N1—H1E	0.8900	C7—H7B	0.9700
O9—C7	1.430 (4)	C19—H19A	0.9600
O9—C6	1.437 (4)	C19—H19B	0.9600
C15—C14	1.396 (4)	C19—H19C	0.9600
C15—C16	1.397 (4)	C8—H8A	0.9700
C15—C19	1.523 (4)	C8—H8B	0.9700
C16—C17	1.393 (5)	C3—C4	1.497 (6)
O6—C1	1.435 (5)	C3—H3A	0.9700
O6—C12	1.440 (4)	C3—H3B	0.9700
O5—C10	1.437 (5)	C5—H5A	0.9700
O5—C11	1.442 (4)	C5—H5B	0.9700
O7—C2	1.445 (5)	C4—H4A	0.9700
O7—C3	1.451 (5)	C4—H4B	0.9700
O10—C9	1.432 (4)	C9—H9A	0.9700
O10—C8	1.440 (4)	C9—H9B	0.9700
C13—C18	1.390 (4)	C12—H12A	0.9700
C13—C14	1.394 (4)	C12—H12B	0.9700
C17—C18	1.393 (4)	C1—C2	1.498 (7)
C17—H17A	0.9300	C1—H1C	0.9700
C18—H18A	0.9300	C1—H1D	0.9700
C14—H14A	0.9300	C2—H2A	0.9700
C11—C12	1.493 (6)	C2—H2B	0.9700
C11—H11A	0.9700	Cl2—O1	1.426 (3)
C11—H11B	0.9700	Cl2—O3	1.439 (3)
C6—C5	1.503 (5)	Cl2—O2	1.441 (3)
C6—H6A	0.9700	Cl2—O4	1.457 (3)
			
C4—O8—C5	114.1 (3)	C15—C19—H19C	109.5
C13—N1—H1A	109.5	H19A—C19—H19C	109.5
C13—N1—H1B	109.5	H19B—C19—H19C	109.5
H1A—N1—H1B	109.5	O10—C8—C7	108.7 (3)
C13—N1—H1E	109.5	O10—C8—H8A	110.0
H1A—N1—H1E	109.5	C7—C8—H8A	110.0
H1B—N1—H1E	109.5	O10—C8—H8B	110.0
C7—O9—C6	111.6 (3)	C7—C8—H8B	110.0
C14—C15—C16	117.0 (3)	H8A—C8—H8B	108.3
C14—C15—C19	120.6 (3)	O7—C3—C4	108.9 (3)
C16—C15—C19	122.3 (3)	O7—C3—H3A	109.9
C17—C16—C15	122.3 (3)	C4—C3—H3A	109.9
C17—C16—Br1	118.3 (2)	O7—C3—H3B	109.9
C15—C16—Br1	119.4 (2)	C4—C3—H3B	109.9
C1—O6—C12	112.7 (3)	H3A—C3—H3B	108.3
C10—O5—C11	112.5 (3)	O8—C5—C6	108.5 (3)
C2—O7—C3	112.0 (3)	O8—C5—H5A	110.0
C9—O10—C8	112.4 (3)	C6—C5—H5A	110.0
C18—C13—C14	120.5 (3)	O8—C5—H5B	110.0
C18—C13—N1	120.1 (3)	C6—C5—H5B	110.0
C14—C13—N1	119.4 (3)	H5A—C5—H5B	108.4
C18—C17—C16	119.5 (3)	O8—C4—C3	108.0 (3)
C18—C17—H17A	120.2	O8—C4—H4A	110.1
C16—C17—H17A	120.2	C3—C4—H4A	110.1
C13—C18—C17	119.2 (3)	O8—C4—H4B	110.1
C13—C18—H18A	120.4	C3—C4—H4B	110.1
C17—C18—H18A	120.4	H4A—C4—H4B	108.4
C13—C14—C15	121.4 (3)	O10—C9—C10	108.9 (3)
C13—C14—H14A	119.3	O10—C9—H9A	109.9
C15—C14—H14A	119.3	C10—C9—H9A	109.9
O5—C11—C12	109.2 (3)	O10—C9—H9B	109.9
O5—C11—H11A	109.8	C10—C9—H9B	109.9
C12—C11—H11A	109.8	H9A—C9—H9B	108.3
O5—C11—H11B	109.8	O6—C12—C11	109.1 (3)
C12—C11—H11B	109.8	O6—C12—H12A	109.9
H11A—C11—H11B	108.3	C11—C12—H12A	109.9
O9—C6—C5	109.3 (3)	O6—C12—H12B	109.9
O9—C6—H6A	109.8	C11—C12—H12B	109.9
C5—C6—H6A	109.8	H12A—C12—H12B	108.3
O9—C6—H6B	109.8	O6—C1—C2	109.9 (4)
C5—C6—H6B	109.8	O6—C1—H1C	109.7
H6A—C6—H6B	108.3	C2—C1—H1C	109.7
O5—C10—C9	109.7 (3)	O6—C1—H1D	109.7
O5—C10—H10A	109.7	C2—C1—H1D	109.7
C9—C10—H10A	109.7	H1C—C1—H1D	108.2
O5—C10—H10B	109.7	O7—C2—C1	109.3 (4)
C9—C10—H10B	109.7	O7—C2—H2A	109.8
H10A—C10—H10B	108.2	C1—C2—H2A	109.8
O9—C7—C8	108.5 (3)	O7—C2—H2B	109.8
O9—C7—H7A	110.0	C1—C2—H2B	109.8
C8—C7—H7A	110.0	H2A—C2—H2B	108.3
O9—C7—H7B	110.0	O1—Cl2—O3	110.3 (2)
C8—C7—H7B	110.0	O1—Cl2—O2	109.5 (2)
H7A—C7—H7B	108.4	O3—Cl2—O2	110.04 (19)
C15—C19—H19A	109.5	O1—Cl2—O4	109.1 (2)
C15—C19—H19B	109.5	O3—Cl2—O4	109.5 (2)
H19A—C19—H19B	109.5	O2—Cl2—O4	108.4 (2)
			
C14—C15—C16—C17	−0.6 (5)	C6—O9—C7—C8	174.6 (3)
C19—C15—C16—C17	179.8 (3)	C9—O10—C8—C7	−178.6 (3)
C14—C15—C16—Br1	177.5 (2)	O9—C7—C8—O10	−67.0 (4)
C19—C15—C16—Br1	−2.1 (4)	C2—O7—C3—C4	169.7 (4)
C15—C16—C17—C18	0.7 (5)	C4—O8—C5—C6	180.0 (3)
Br1—C16—C17—C18	−177.4 (3)	O9—C6—C5—O8	66.3 (4)
C14—C13—C18—C17	−0.5 (5)	C5—O8—C4—C3	−164.9 (3)
N1—C13—C18—C17	179.1 (3)	O7—C3—C4—O8	−66.0 (4)
C16—C17—C18—C13	−0.2 (5)	C8—O10—C9—C10	169.2 (3)
C18—C13—C14—C15	0.6 (5)	O5—C10—C9—O10	68.3 (4)
N1—C13—C14—C15	−178.9 (3)	C1—O6—C12—C11	−175.7 (4)
C16—C15—C14—C13	−0.1 (5)	O5—C11—C12—O6	−60.0 (4)
C19—C15—C14—C13	179.6 (3)	C12—O6—C1—C2	173.7 (4)
C10—O5—C11—C12	178.1 (3)	C3—O7—C2—C1	177.2 (4)
C7—O9—C6—C5	180.0 (3)	O6—C1—C2—O7	67.1 (5)
C11—O5—C10—C9	−174.4 (3)		

### Hydrogen-bond geometry (Å, °)

**Table:**

*D*—H···*A*	*D*—H	H···*A*	*D*···*A*	*D*—H···*A*
N1—H1B···O5	0.89	2.19	2.955 (4)	144
N1—H1E···O6	0.89	2.29	2.970 (4)	133
N1—H1E···O7	0.89	2.12	2.893 (4)	145
N1—H1A···O8	0.89	2.22	2.905 (4)	134
N1—H1A···O9	0.89	2.19	2.966 (4)	145
N1—H1B···O10	0.89	2.22	2.912 (4)	134
